# Treosulfan-Versus Melphalan-Based Reduced Intensity Conditioning in HLA-Haploidentical Transplantation for Patients ≥ 50 Years with Advanced MDS/AML

**DOI:** 10.3390/cancers16162859

**Published:** 2024-08-16

**Authors:** Alessia Fraccaroli, Elena Stauffer, Sarah Haebe, Dusan Prevalsek, Lena Weiss, Klara Dorman, Heidrun Drolle, Michael von Bergwelt-Baildon, Hans-Joachim Stemmler, Tobias Herold, Johanna Tischer

**Affiliations:** 1 Hematopoietic Stem Cell Transplantation Unit, Department of Medicine III, LMU University Hospital, 81377 Munich, Germany; alessia.fraccaroli@med.uni-muenchen.de (A.F.); e.stauffer@med.uni-muenchen.de (E.S.); sarah.haebe@med.uni-muenchen.de (S.H.); dusan.prevalsek@med.uni-muenchen.de (D.P.); lena.weiss@med.uni-muenchen.de (L.W.); klara.dorman@med.uni-muenchen.de (K.D.); heidrun.drolle@med.uni-muenchen.de (H.D.); michael.bergwelt@med.uni-muenchen.de (M.v.B.-B.); joachim.stemmler@med.uni-muenchen.de (H.-J.S.); tobias.herold@med.uni-muenchen.de (T.H.); 2 German Cancer Consortium (DKTK), Partner Site Munich, a Partnership between the DKFZ Heidelberg and the University Hospital of the LMU, 81377 Munich, Germany

**Keywords:** treosulfan, melphalan, AML, MDS, HLA-haploidentical transplantation, sequential therapy

## Abstract

**Simple Summary:**

Relapse and treatment-related side effects pose significant challenges for older patients with high-risk blood cancers (myelodysplastic syndrome (MDS)/acute myeloid leukemia (AML)) undergoing hematopoietic stem cell transplants from HLA-haploidentical donors. We compared two preparative treatment regimens to identify the most tolerable and effective approach: one using fludarabine, cyclophosphamide, and melphalan, and the other using fludarabine, cyclophosphamide, and treosulfan. Our goal was to determine which treatment offers better survival rates and fewer side effects. We found that both regimens resulted in similar survival outcomes. However, the melphalan regimen was associated with fewer relapses but more treatment-related deaths, while the treosulfan regimen had fewer side effects but exhibited poorer disease control. These findings suggest that treosulfan may be safer, though higher doses might improve disease control, providing valuable insights for future treatments.

**Abstract:**

Relapse and regimen-related toxicities remain major challenges in achieving long-term survival, particularly among older patients with high-risk myelodysplastic syndrome (MDS) or acute myeloid leukemia (AML) undergoing allogeneic hematopoietic stem cell transplantation (allo-HSCT). Previous studies have demonstrated the feasibility of treosulfan-based conditioning, noting stable engraftment and low non-relapse mortality (NRM) in patients undergoing HLA-matched allo-HSCT. However, data on treosulfan-based conditioning in the HLA-haploidentical transplantation (HaploT) setting are limited. We retrospectively compared conditioning with fludarabine–cyclophosphamide (FC)–melphalan (110 mg/m^2^) and FC-treosulfan (30 g/m^2^) prior to HaploT using post-transplantation cyclophosphamide (PTCy) in patients with high-risk MDS/AML patients ≥ 50 years, transplanted from 2009–2021 at our institution (*n* = 80). After balancing patient characteristics by a matched-pair analysis, we identified twenty-one matched pairs. Two-year OS and LFS were similar among the groups (OS 66% and LFS 66%, *p* = 0.8 and *p* = 0.57). However, FC-melphalan was associated with a significantly lower probability of relapse compared to FC-treosulfan (0% vs. 24%, *p* = 0.006), counterbalanced by a higher NRM (33% vs. 10%, *p* = 0.05). Time to engraftment and incidences of acute and chronic graft-versus-host disease (GvHD) did not differ significantly. In conclusion, HaploT using FC-treosulfan in combination with PTCy in patients aged ≥50 years with MDS/AML appears safe and effective, particularly in advanced disease stages. We confirm the favorable extramedullary toxicity profile, allowing for potential dose intensification to enhance antileukemic activity.

## 1. Introduction

HLA-haploidentical hematopoietic stem cell transplantation (HaploT) with post-transplantation cyclophosphamide (PTCy) [1] became a valuable alternative for elderly patients who often lack fully matched related donors and require time-critical allogeneic hematopoietic stem cell transplantation (allo-HSCT) due to aggressive disease.

Numerous studies have demonstrated its feasibility and low toxicity profile even when combined with higher intensity conditioning or sequential therapy concepts. This has resulted in long-term disease control in patients transplanted with active disease, with graft-versus-host disease (GvHD) rates not exceeding the ones for remission patients [2,3,4,5,6]. However, no standard conditioning in unmanipulated HaploT for older age high-risk (HR) myelodysplastic syndrome/acute myeloid leukemia (MDS/AML) patients has yet been defined. 

Standard conditioning regimens prior to allo-HSCT are often associated with a considerable risk of severe adverse events, especially in elderly patients suffering from HR MDS/AML [7,8]. Recently, efforts have focused on redesigning conditioning regimens into less toxic approaches, while maintaining their high efficacy. Based on the emerging data of a large randomized prospective phase III trial showing superiority of fludarabine and treosulfan (FluTreo) over the traditional fludarabine and busulfan (FluBu) regimens in older MDS/AML patients transplanted in remission [9,10] and large registry studies confirming those observations [11,12,13,14,15,16], we incorporated treosulfan-based conditioning into our clinical routine to further optimize HaploT feasibility in older age MDS/AML patients, with the aim of reducing transplantation-related mortality (NRM) while maintaining the antileukemic activity.

Up to now, data for treosulfan-based conditioning in the unmanipulated HaploT setting in HR AML/MDS patients have been limited. Here, we aimed to investigate the impact of treosulfan-based (FC-Treo) versus melphalan-based (FC-Mel) conditioning in patients ≥ 50 years old, with HR AML/MDS undergoing HaploT through a matched-pair analysis, focusing on feasibility, toxicity, and disease control.

## 2. Patients and Methods

This is a single-center retrospective observational study on patients undergoing HaploT at our center aiming to assess the efficacy and safety of treosulfan-based (3 × 10 g/m^2^) compared to melphalan-based (110 mg/m^2^) conditioning in patients ≥ 50 years old. Data analysis consisted of retrospective chart review. The study was conducted in accordance with the German legislation and the revised Helsinki Declaration and evaluated and approved by the local ethics committee of the Ludwig-Maximilians University of Munich.

**Figure 1:**
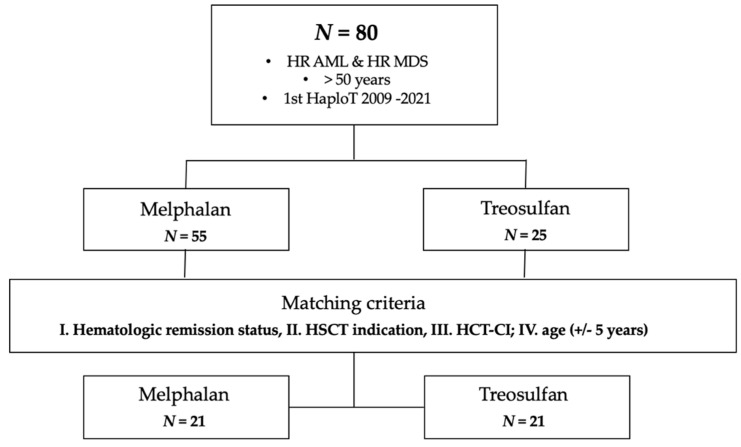
CONSORT diagram. Abbreviations: AML, acute myeloid leukemia, HaploT, HLA-haploidentical hematopoietic stem cell transplantation; HCT-CI, hematopoietic cell transplantation-specific comorbidity index, HR, high-risk, HSCT, hematopoietic stem cell transplantation, MDS, myelodysplastic syndrome.

All consecutive patients ≥ 50 years old who fulfilled the following criteria were included: (A) diagnosis of HR AML, as defined by either primary refractory or relapsed AML, secondary AML, or AML harboring genetic aberrations classified as adverse according to ELN 2017 [17], (B) HR-MDS IPSS-R > 4.5 [18], (C) undergoing a first HaploT between 2009 and 2021, (D) with a conditioning regimen including either treosulfan (30 g/m^2^) or melphalan (110 mg/m^2^). Among the eighty patients identified (treosulfan *n* = 25; melphalan *n* = 55), a matched-pair analysis using the following predefined matching criteria in descending hierarchical order was performed: (1) hematologic remission status (≥5% blast yes or no) at start of conditioning, (2) transplantation indication (relapse, refractory, HR genetic aberrations in first CR at start of conditioning), (3) hematopoietic cell transplantation-specific comorbidity index (HCT-CI), and (4) age (+/−5 y) in order to compare outcomes among the different conditioning drugs (Figure 1). Twenty-one patients undergoing treosulfan-based conditioning could be successfully pair-matched with twenty-one patients treated with melphalan-based conditioning. Nine patients were included in a previous publication [3].

### 2.1. Treatment

Sequential conditioning consisted of either FLAMSA (fludarabine 30 mg/m^2^, cytarabine 2 g/m^2^, amsacrine 100 mg/m^2^ intravenously (iv) over 4 days) or clofarabine (30 mg/m^2^ iv over 5 days). The cytarabine dose was reduced to 1 g/m^2^ in patients above the age of 60 years. Following a three-day break, conditioning comprised fludarabine 30 mg/m^2^ for five days and cyclophosphamide 14.5 mg/kg for two days combined with either melphalan 110 mg/m^2^ (day −1) or treosulfan 10 g/m^2^ per day from day −3 to −1 (Supplementary Figure S1). The decision to apply sequential conditioning was based on patient- and disease-specific features, including comorbidities, previous treatments, prior response to therapy, and disease remission status according to our local center strategy. Clofarabine was preferred over FLAMSA in patients who underwent several courses of cytarabine-based therapy before relapse Figure S1.

PTCy was applied as previously described [1] at a dose of 50 mg/kg per day at day +3 and +4 after unmanipulated HaploT. Post-grafting immunosuppression was uniformly performed using a combination of tacrolimus and mycophenolate mofetil (MMF), starting from day +5, tapered from day +120, and discontinued by day +140 in case of tacrolimus and discontinuation at day +35 for MMF, if no signs of GvHD were present. In the absence of history or evidence of GvHD prophylactic donor lymphocyte infusion (pDLI) was evaluated after discontinuation of immunosuppression. Supportive care included standard antimicrobial prophylaxis (micafungin or caspofungin) and infection surveillance according to local center strategies [3].

### 2.2. Definitions and Evaluation of Response

Refractoriness was defined as primary induction failure (PIF) with patients not achieving complete remission (CR) after receiving at least two courses of intensive induction chemotherapy containing high-dose cytarabine or relapse refractory to salvage treatment. Upfront transplantation was used in untreated HR MDS (EB-2 or adverse cytogenetics). Relapse was defined by bone marrow (BM) blast counts of >5%, extramedullary manifestation, or the recurrence of leukemic blasts in peripheral blood. Engraftment was defined following the Center for International Blood and Marrow Transplant Research criteria (CIBMTR forms manual, 2014). Hematopoietic chimerism of BM was assessed using either fluorescent in situ hybridization (FISH) or quantitative polymerase chain reaction (qPCR) of variable nucleotide tandem repeats (VNTRs) and short tandem repeats (STRs). Conditioning intensity was defined as previously described by [19].

Acute and chronic GvHDs were graded according to Keystone criteria [20] and National Institutes of Health (NIH) consensus criteria [21], respectively.

National Cancer Institute Common Terminology Criteria for Adverse Events (NCI CTC AE version 3.0) were used for non-hematologic toxicity assessment starting from sequential therapy initiation until day +30. 

### 2.3. Statistical Analysis

Patient characteristics were compared by using a Kruskal–Wallis test for quantitative variables and a X^2^ or Fisher’s exact test for categorical variables. All endpoints were measured from the time of transplantation. The study endpoints were overall survival (OS), leukemia-free survival (LFS), relapse incidence (RI), NRM, engraftment, acute GvHD, chronic GvHD, and hematologic recovery. NRM and relapse events were considered as competing risks. Probabilities of OS and LFS were estimated by using the Kaplan–Meier method and compared by using the log-rank test. Estimates of NRM, relapse rate, and chronic GvHD were calculated by using cumulative incidence curves to accommodate competing risks and were compared by using Gray’s test. 

Survival probabilities are presented as percentages and 95% confidence intervals (CIs). All tests were two-sided and *p* values < 0.05 were considered significant. Analyses were performed using SPSS for Windows version 20.0 (SPSS, Chicago, IL, USA) and R Project software, version 2.15.2.

## 3. Results

### 3.1. Patient, Donor, and Transplant Characteristics

A total of twenty-five patients with HR MDS/AML, who were ≥50 years old and underwent unmanipulated HaploT between 2009 and 2021 at our center using treosulfan-based (3 × 10 g/m^2^) conditioning, were considered for potential matching with recipients of a melphalan-based (1 × 110 mg/m^2^) regimen (*n* = 55). Twenty-one patients were successfully pair-matched with patients undergoing melphalan-based conditioning (*n* = 21) according to the matching strategy mentioned above: (1) remission status: *p* = 1.0; (2) allo-HSCT indication: *p* = 1.0; (3), hematopoietic cell transplantation (HCT)-specific comorbidity index (HCT-CI) score: *p* = 1.0; (4) age: *p* = 0.65. Each group consisted of seventeen AML patients and four MDS patients, respectively. The majority of patients (62%) presented with active disease. The other collected patient and disease characteristics were well balanced and showed no significant difference between the two groups. Details on patient, donor, and transplant characteristics are provided in Table 1.

### 3.2. Engraftment and Chimerism

Median time to neutrophil engraftment did not significantly differ between the groups although BM was more frequently used as a graft source in treosulfan-treated patients (FC-Mel: 19 days, FC-Treo: 19 days; *p* = 0.89). Five patients within the melphalan-treated cohort—all undergoing sequential conditioning and suffering from active disease before allo-HSCT—were censored due to death in early aplasia (*n* = 4) and graft rejection due to aspergillus sepsis (*n* = 1); all treosulfan-treated patients were engrafted. Median time to platelet engraftment was delayed in the melphalan-treated group (median: 31 days) compared to the treosulfan group (median: 26 days) (*p* = 0.05).

By day +30 after HaploT, unselected BM donor chimerism > 95% was detected in the majority of patients (33/34) regardless of the chosen conditioning regimen. Chimerism data were not available in three patients.

### 3.3. Regimen-Related Toxicities and Infections

Within the observation period (start of conditioning to day +30) 24 of 42 patients (57%) experienced at least one grade III or IV non-hematologic adverse event. The latter included diarrhea, fever, renal toxicity, mucositis, and liver toxicity, listed in order of frequency (Table 2). Seven patients (FC-Mel: *n* = 6; FC-Treo: *n* = 1) presented with more than one higher-grade toxicity.

The frequency and severity of all adverse events did not reveal any dependency on the alkylating agent used, except for the occurrence of diarrhea, which was more frequently seen in FC-Mel-treated patients (*p* = 0.02). In most of the patients affected (90%), diarrhea started or worsened with the application of PTCy. No veno-occlusive disease occurred. In three patients (FC-Mel *n* = 1; FC-Treo *n* = 2) transplantation-associated microangiopathy (TA-TMA) with moderate disease course was diagnosed. All except two patients developed fever throughout transplantation with sterile blood cultures. Fever resolved after the start of post-grafting immunosuppression. One Epstein–Barr Virus (EBV) reactivation occurred and was treated with rituximab. No post-transplantation lymphoproliferative disorder was diagnosed. Cytomegalovirus (CMV) reactivation was diagnosed in 7/15 and 11/13 patients at risk in the FC-Mel and FC-Treo groups, respectively (*p* = 0.05). Three cases of BK-virus-associated hemorrhagic cystitis were observed. Invasive fungal infections were observed in eight patients, including cases of invasive aspergillosis (FC-Mel *n* = 3; FC-Treo *n* = 1) and fungemia (Candida spp) (FC-Mel *n* = 4).

### 3.4. Graft-Versus-Host Disease

The cumulative incidence (CI) of grade II–IV acute GvHD at day +100 revealed no significant difference between FC-Mel- and FC-Treo-treated patients and was 24% (95%CI: 19–29) and 14% (95%CI: 11–17), respectively (*p* = 0.79). Seventeen out of the twenty-two patients (77%) affected by acute GvHD exhibited exclusive cutaneous manifestations. No liver involvement was seen. 

One-year CI of chronic GvHD ≥ moderate reached 14% (95%CI: 11–17) in the FC-Mel group vs. 10% (95%CI: 8–12) in the FC-Treo (*p* = 0.46) group, with only one noted case of severe chronic GvHD involving skin and lung (bronchiolitis obliterans syndrome) in the FC-Mel group.

### 3.5. Outcome, Non-Relapse Mortality, and Relapse

**Figure 2:**
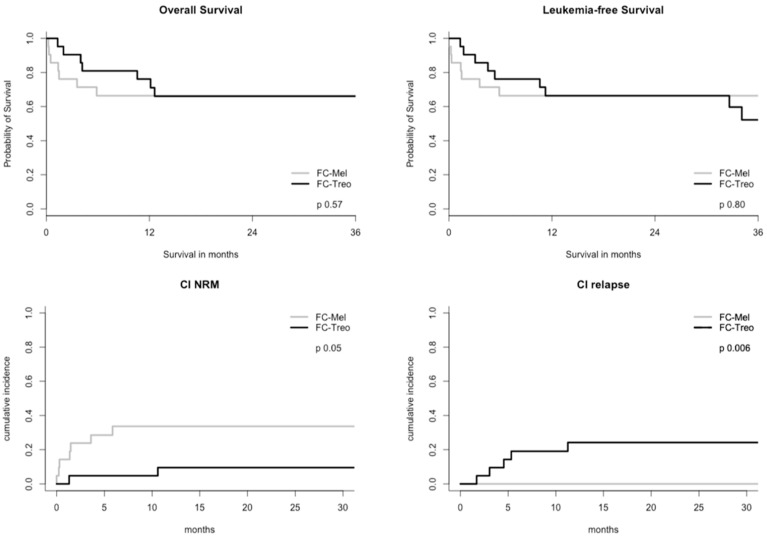
Comparison of transplant outcome following FC-Mel and FC-Treo conditioning regimens ahead of HaploT. Abbreviations: CI, cumulative incidence; FC, combination of fludarabine and cyclophosphamide; HaploT, HLA-haploidentical hematopoietic stem cell transplantation; Mel, melphalan; NRM, non-relapse mortality; Treo, treosulfan.

With a median follow-up of survivors of 3.3 years (5 months–12.3 years), the estimated two-year OS was 66% (95%CI: 53–82) for the entire cohort. Similarly, two-year LFS was 66% (95% CI: 53–82), with more than half of the patients achieving a durable remission after HaploT, regardless of conditioning regimen used (OS: FC-Treo: 2y 66% (95%CI 0.49–0.90) FC-Mel: 2y 66% (95%CI 0.49–0.90), HR 1.13, *p* = 0.8; LFS: FC-Treo: 1y 66% (95%CI 0.48–0.90), FC-Mel: 1y 66% (95%CI 0.49–0.90), HR 1.35, *p* = 0.57) (Figure 2). Nonetheless, both cohorts showed a notable difference regarding NRM (*p* = 0.05) and incidence of relapse (*p* = 0.006). The two-year CI of NRM was 33% vs. 10% for the FC-Mel- and FC-Treo-treated patients, respectively. Causes of NRM comprised infections in five patients (FC-Mel) and organ toxicity in two (FC-Mel) and one patient (FC-Treo) as well as GvHD in one patient in each cohort. Univariate analysis showed a non-significant trend towards better OS and LFS according to remission status ahead of HaploT (2y OS: CR 82% vs. active disease 56%, HR 2.74, *p* = 0.06; 2y LFS: CR 82% vs. active disease 56%, HR 2.17, *p* = 0.13). There was no significant difference in CI of NRM (*p* = 0.79) or CI of relapse (*p* = 0.29), Figure S2.

The most common cause of death in the treosulfan-treated patients was relapse, reflected in a CI of relapse at two years of 24% vs. 0% in the FC-Treo vs. FC-Mel group, respectively. Relapse affected only two patients in the FC-Mel group and occurred after a median of 5 years, with one patient opting for combination therapy using azacitidine/venetoclax and achieving CR, while the other declined further treatment. In the FC-Treo cohort, seven patients experienced relapse, three of whom encountered early relapse before day +150 with aggressive disease biology. The remaining individuals underwent rescue treatment, including azacitidine ± venetoclax or targeted therapy with enasidenib, resulting in a best response of partial remission. Notably, a higher proportion of FC-Treo vs. FC-Mel patients (seven vs. three) received prophylactic donor lymphocyte infusion (pDLI). Patients with a history of GvHD (all grades) were excluded from pDLI application in our haplo-cohort due to safety concerns. Details are reported in Figure 2 and Table 3.

## 4. Discussion

In this study, we assessed the feasibility, toxicity, and efficacy of a treosulfan-based conditioning (FC-Treo 30 g/m^2^) regimen within the context of unmanipulated HaploT using PTCy as GvHD prophylaxis for HR MDS/AML patients ≥ 50 years of age. The preparative regimen was intensified using either FLAMSA or clofarabine due to HR disease; specifically, 62% of patients suffered from active disease at the start of conditioning. Using a matched-pair comparison with melphalan-based conditioning (FC-Mel 110 mg/m^2^), we found no significant differences in terms of LFS and OS (2y OS and LFS 66%, *p* = 0.8 for OS, *p* = 0.57 for LFS) between the regimens. Yet, FC-Treo demonstrated a higher incidence of relapse (24% vs. 0%, *p* = 0.006) and lower NRM (10% vs. 33%, *p* = 0.05) compared to the patients treated with FC-Mel, suggesting an inferior antileukemic effect but likewise lower toxicity. 

Our findings are consistent with previous studies. Recently, a large EBMT analysis compared outcomes after myeloablative conditioning (MAC) with either fludarabine–melphalan (FluMel 140 mg/m^2^) or fludarabine–treosulfan (FluTreo 42 g/m^2^) ahead of HLA-matched allo-HSCT and observed better antileukemic activity in the FluMel arm (CI relapse at 3 years 32.4 vs. 40.5%, *p* = 0.001) at the expense of higher toxicity (CI NRM at 3 years 25.7% vs. 20.2%, *p* = 0.06) [22]. Similarly to our data these differences did not translate into a survival benefit (3y OS 54% vs. 51.2%, *p* = 0.49). It is worth noting that in contrast to our study, different in vivo T-cell depletion strategies were used, and HaploT as well as patients with active disease were excluded from the analysis. Interestingly, the relapse incidence of 24% in our HaploT-FC-Treo cohort is similar to the HLA-matched-FluTreo arm (24.6%) of a randomized registration trial [9,10]. This latter trial, MC-FludT.14/L, demonstrated statistically significant non-inferiority for treosulfan compared to busulfan, along with a clinically relevant improvement in event-free survival (EFS) for treosulfan-treated AML and MDS patients > 50 years of age or with an HCT-CI score of >2. However, it is important to note that the study performed by Beelen and colleagues [9,10], in contrast to ours, predominantly included AML patients in CR or MDS patients with a lower relapse risk. While the treosulfan dose (30 g/m^2^) used by Beelen and colleagues was consistent with our study, we intensified RIC by the addition of FLAMSA or clofarabine to enhance the antileukemic activity of the regimen [3,5,23,24]. 

The first to combine FLAMSA with treosulfan (30 g/m^2^) in the HLA-matched setting was the Cologne group [25,26,27]. In contrast to our data, Holtick et al. observed a higher toxicity of treosulfan (CI NRM 28%) compared to 4Gy TBI (13%), probably due to the patient characteristic imbalances, i.e., TBI-treated patients were significantly younger (*p* < 0.001) with fewer comorbidities (*p* = 0.005). However, their sequential approach with treosulfan-RIC demonstrated promising efficacy, showing stable engraftment and favorable survival (4 year 47% FLAMSA/TBI vs. 43% FLAMSA/Treo). Notably, in the subgroup of non-remission patients treosulfan achieved better results in terms of disease control (CI relapse 70% TBI vs. 35% treosulfan, *p* = 0.069). In a subsequent study from the same group focusing on refractory AML, they could not confirm the differences between FLAMSA-TBI and FLAMSA-treosulfan in terms of relapse incidence (29% at 3 years) and survival (OS 61% at 3 years) [27]. Results from this study align with our data presented here. 

To our knowledge this study represents the first to combine FLAMSA with treosulfan-RIC in any transplantation setting, including HaploT, for older age HR AML/MDS patients. Similar approaches to enhance the antileukemic efficacy of treosulfan beyond its dose alteration were pursued by (1) O’Hagan Henderson et al., who combined high-dose cytarabine with a FluTreo MAC in patients with poor-risk myeloid disease (active disease 55%) [28], and (2) Shargian-Alon et al. who combined FLAG-IDA with treosulfan conditioning in older age (median age 64 years), HR AML/MDS patients. Compared to our smaller FC-Treo cohort, the non-hematological toxicities described by O’Hagan Henderson [28] were greater in percentage terms. Our predominant toxicity was diarrhea, likely due to high-dose PTCy application for GvHD prophylaxis in the HaploT setting. The liver toxicity which affected 84% of the high-dose cytarabine patients did not transfer to our results. Yet, it must be stated that similar doses of cytarabine are administered in the FLAMSA protocol; only a minority of our patients received clofarabine (*n* = 3) or no cytoreduction (*n* = 3). However, in the melphalan group infection was the leading cause of mortality. Interestingly, the other sequential studies with treosulfan backbone found a similar result [27,28]. In contrast, we did not find any lethal or early infections in the FLAMSA-FC-Treo group, despite similar neutrophil engraftment.

So far, limited data are available regarding the utilization of treosulfan in the context of HaploT. A subgroup comprising 67 AML patients in different remission states was identified within a comprehensive Italian registry study. Detailed survival and feasibility data specific to this subgroup as well as a specification of T-cell depletion were not provided [29]. A recent EBMT analysis underpins these results for patients with AML in remission and further demonstrates a similar efficacy to the TBF regimen [30].

Incidences of acute and chronic GvHD did not differ between the regimens and are rather low considering the HR cohort with mainly active disease [31]. Treosulfan is known for its high immunosuppressive properties [32] and has been associated with lower GvHD rates compared to the busulfan-based regimens in patients transplanted in remission [12]. In line with our data, this finding could not be confirmed in patients with persistent leukemia [33]. With a CI of chronic GvHD (≥moderate) of 10%, GvHD rates are rather low compared to similar studies. When interpreting the results, it should be considered that we present data on unmanipulated HaploT with the use of PTCy as GvHD prophylaxis and cumulatively higher doses of fludarabine, inherent of the FLAMSA FC protocol. Furthermore, given the comparable GvHD incidences, it is unlikely that the FC-Mel regimen exerts a more pronounced GvL effect, thereby contributing to the lower CI of relapse. One might assume that treosulfan’s inherent antileukemic property in the HaploT context is less, if confronted with patients with adverse or active AML and used at a dose of 30 g/m^2^. 

Historically, different treosulfan dose intensities have been tested and a total dose of 42 g/m^2^ seems to show the highest efficacy [15,32]. A dose reduction to 30 g/m^2^ does not appear to do any less in AML patients transplanted in CR1, especially when of older age, due to a substantial reduction of NRM [9,10]. The favorable extramedullary toxicity profile in conjunction with the lack of dose-limiting toxicities leads us to the assumption that a treosulfan dose escalation should be considered in patients >50 years of age suffering from HR MDS/AML. Based on this premise further investigations should focus on finding the optimal balance between antileukemic efficacy and toxicity in HaploT with treosulfan-based conditioning.

Due to the rather small numbers in the present single-center study, we were not able to perform a multivariate analysis on features predicting transplant outcome in the setting of HR MDS/AML. Further limitations of the present analysis are mostly related to its retrospective design with an ongoing patient selection bias, which may have affected data interpretation. However, in comparison to register analysis, our study provides an accurate and detailed comparison, especially regarding GvHD, infection, and treatment-related toxicity analysis, not impacted by institutional expertise or different local standards.

## 5. Conclusions

In conclusion, we confirmed our hypothesis that treosulfan-based conditioning in unmanipulated HaploT with PTCy for MDS/AML patients > 50 years old improves the feasibility of this approach by reducing NRM. It is safe and well tolerated, resulting in stable engraftment, favorable toxicity profile, with no severe GvHD. Despite the similar outcomes in terms of OS and LFS, both cohorts showed notable differences regarding CIs of NRM and relapse. Treosulfan- and PTCy-based HaploT in older HR MDS and AML patients with advanced disease shows lower NRM but higher relapse rates compared to a melphalan-based approach. Despite the limitations of our retrospective single-center study, we suggest that treosulfan-based intensified RIC in HaploT might be a valuable alternative in elderly patients. However, its intensity should be reconsidered in this specific patient cohort and deserves prospective evaluation. Furthermore, our data indicate that older age should not be taken as a criterion to withhold transplant in patients with active AML.

## Figures and Tables

**Table 1:** Patient and transplant baseline characteristics.

Characteristics	No. (%)			
	All	By Conditioning Regimen		
		FC-Melphalan	FC-Treosulfan	*p*-Value
Total patients	42 (100)	21 (100)	21 (100)	
Patient age, years *				0.65
Median	65	65	65	
Range	53–75	53–74	57–75	
Patient sex				0.52
Male	27 (64)	12 (57)	15 (71)	
Female	15 (36)	9 (43)	6 (19)	
Diagnosis *				1.0
AML	34 (81)	17 (81)	17 (81)	
De novo	16 (38)	6 (29)	10 (48)	
MDS	8 (19)	4 (19)	4 (19)	
HSCT indication *				1.0
PIF	8 (19)	4 (19)	4 (19)	
relapse	6 (14)	3 (14)	3 (14)	
HR-cytogenetics	18 (43)	9 (43)	9 (43)	
Untreated ^§^	10 (24)	5(24)	5 (24)	
Status of remission *				1.0
Non-remission ^#^	26 (62)	13 (62)	13 (62)	
CR	16 (38)	8 (38)	8 (38)	
Modified DRI *				0.83
Intermediate risk	14 (33)	7 (33)	7 (33)	
High risk	25 (60)	12 (57)	13 (62)	
Very high risk	3 (7)	2 (10)	1 (5)	
HCT-CI score				1.0
0–2	23 (55)	12 (57)	11 (52)	
>2	19 (45)	9 (43)	10 (48)	
Donor age, years				0.56
Median	37.5	39	36	
Range	21–64	21–64	19–63	
Donor sex				0.73
Male	31 (74)	16 (76)	15 (71)	
Female	11 (26)	5 (24)	6 (29)	
Donor sex match				0.33
Patient male/donor female	6 (14)	3 (14)	3 (14)	
Patient female/donor male	10 (24)	7 (33)	3 (14)	
Match	26 (62)	11 (52)	15 (71)	
ABO match	22 (52)	13 (62)	9 (43)	0.22
CMV match	28 (67)	13 (62)	15 (71)	0.51
Cytoreduction prior to conditioning				0.11
FLAMSA	35 (83)	15 (71)	20 (95)	
Clofarabin	4 (10)	4 (19)	0 (0)	
No cytoreduction	3 (7)	2 (10)	1 (5)	
Stem cell source				0.20
BM	27 (64)	12 (57)	15 (71)	
PBSC	15 (36)	9 (43)	6 (29)	
Median cell dose (range)				n.a
NC × 10^8^/kg BW	2.7 (1.6–4.4)	2.95 (1.8–4.4)	2.6 (1.6–4.0)	
CD34 × 10^6^/kg BW	6.97 (4–9.4)	6.2 (4–9.4)	9.4 (4.3–11.65)	
GvHD prophylaxis				1.0
PTCy-Tac-MMF	42 (100)	21 (100)	21 (100)	
Year of transplant				0.27
Median	2018	2014	2018	
Range	2009–2021	2009–2021	2016–2021	
Median FU, years	3.3 (5 mo–12.3 yr)	6.2 (5 mo–12.3 yr)	3.1 (11 mo–4.2 yr)	0.13

* Served as matching criteria. ^§^ Including all 8 MDS patients, 4 in each group. ^#^ MDS patients (*n* = 8; *n* = 4 in FC-melphalan, *n* = 4 in FC-treosulfan), AML patients (*n* = 34; *n* = 17 in FC-melphalan, *n* = 17 in FC-treosulfan). Abbreviations: FC, combination of fludarabine and cyclophosphamide; AML, acute myeloid leukemia; tAML, therapy-associated acute myeloid leukemia; MPN, myeloproliferative neoplasm; MDS, myelodysplastic syndrome; PIF, primary induction failure; CR, complete remission; DRI, disease risk index; HCT-CI, hematopoietic cell transplantation-specific comorbidity index; No., number; HLA, human leukocyte antigen; CMV, cytomegalovirus; BM, bone marrow; PBSC, peripheral blood stem cell; NC, nucleated cell; kg, kilogram; BW, body weight; RIC, reduced intensity conditioning; PTCy, post-transplant cyclophosphamide; MMF, mycophenolate mofetil; Tac, tacrolimus; FU, follow-up.

**Table 2:** Frequency of all CTCAE grade III–IV adverse events.

CTCAE Category	FC-Melphalan		FC-Treosulfan		
	III°	IV°	III°	IV°	*p*-Value
No. of patients with any event (%)	9 (43)	7 (33)	6 (29)	3 (14)	
GI tract					
Mucositis	1 (5)	1 (5)	0 (0)	1 (5)	*n.s*.
Nausea and vomiting	0 (0)	0 (0)	0 (0)	0 (0)	*n.s*.
Diarrhea	9 (43) *	3 (14)	3 (14)	2 (10)	**0.03**
Hyperbilirubinemia	1 (5) ^#^	0 (0)	0 (0)	0 (0)	*n.s*.
Elevated transaminases	1 (5) ^#^	1 (5)	0 (0)	0 (0)	*n.s*.
Renal failure					
Creatinine elevation	1 (5)	1 (5)	0 (0)	0 (0)	*n.s*.
Hemodialysis	1 (5)	1 (5)	0 (0)	0 (0)	*n.s*.
Hemorrhagic cystitis	2 (10)	0 (5)	1 (5)	0 (0)	*n.s*.
Skin					
Rash	0 (0)	0 (0)	0 (0)	0 (0)	*n.s.*
Hand–foot syndrome	0 (0)	0 (0)	0 (0)	0 (0)	*n.s.*
Cardiovascular system					
Arrhythmia	0 (0)	0 (0)	1 (5)	0 (0)	*n.s.*
CNS					
Paresthesia	0 (0)	0 (0)	0 (0)	0 (0)	*n.s.*
Confusion	0 (0)	0 (0)	0 (0)	0 (0)	*n.s.*
Fever	3 (14)	1 (5)	4 (19)	0 (0)	*n.s.*

* Including two patients who underwent clofarabin pretreatment. ^#^ Including one patient who underwent clofarabin pretreatment. Abbreviations: CNS, central nervous system; CTCAE, Common Terminology Criteria for Adverse Events; FC, combination of fludarabine and cyclophosphamide; GI, gastrointestinal; No., number.

**Table 3:** Causes of death.

Primary Cause of Death	No. (%)		
	All	By Conditioning Regimen	
		FC-Melphalan	FC-Treosulfan
Relapse	9 (21)	2 (10)	7 (33)
Progressive disease ^†^	0 (0)	0 (0)	0 (0)
NRM ^‡^	11 (26)	9 (42)	2 (10)
Infection	3	3	0
GvHD	1	1	0
GvHD and infection	1	0	1
Graft rejection	1	1	0
Bleeding	1	0	1
Organ toxicity	2	2	0
Organ toxicity and infections	2	2	0

^†^ Defined as disease persistence/early disease progression before day +30. ^‡^ For more information see text. Abbreviations: NRM, non-relapse mortality; GvHD, graft-versus-host disease.

## Data Availability

Data used in this work are available upon reasonable request from the corresponding author.

## Supplementary Materials

The following supporting information can be downloaded at: https://www.mdpi.com/article/10.3390/cancers16162859/s1, Figure S1: Schematic representing sequential conditioning. Figure S2: Comparison of transplant outcome according to remission status ahead of HaploT. Abbreviations: CI, cumulative incidence; CR complete remission.
