# Supplementary figures and images for: Treosulfan-Versus Melphalan-Based Reduced Intensity Conditioning in HLA-Haploidentical Transplantation for Patients ≥ 50 Years with Advanced MDS/AML
Source: Cancers (Basel). 2024 Aug 16;16(16):2859. doi: 10.3390/cancers16162859 (PMC11353158; doi:10.3390/cancers16162859)

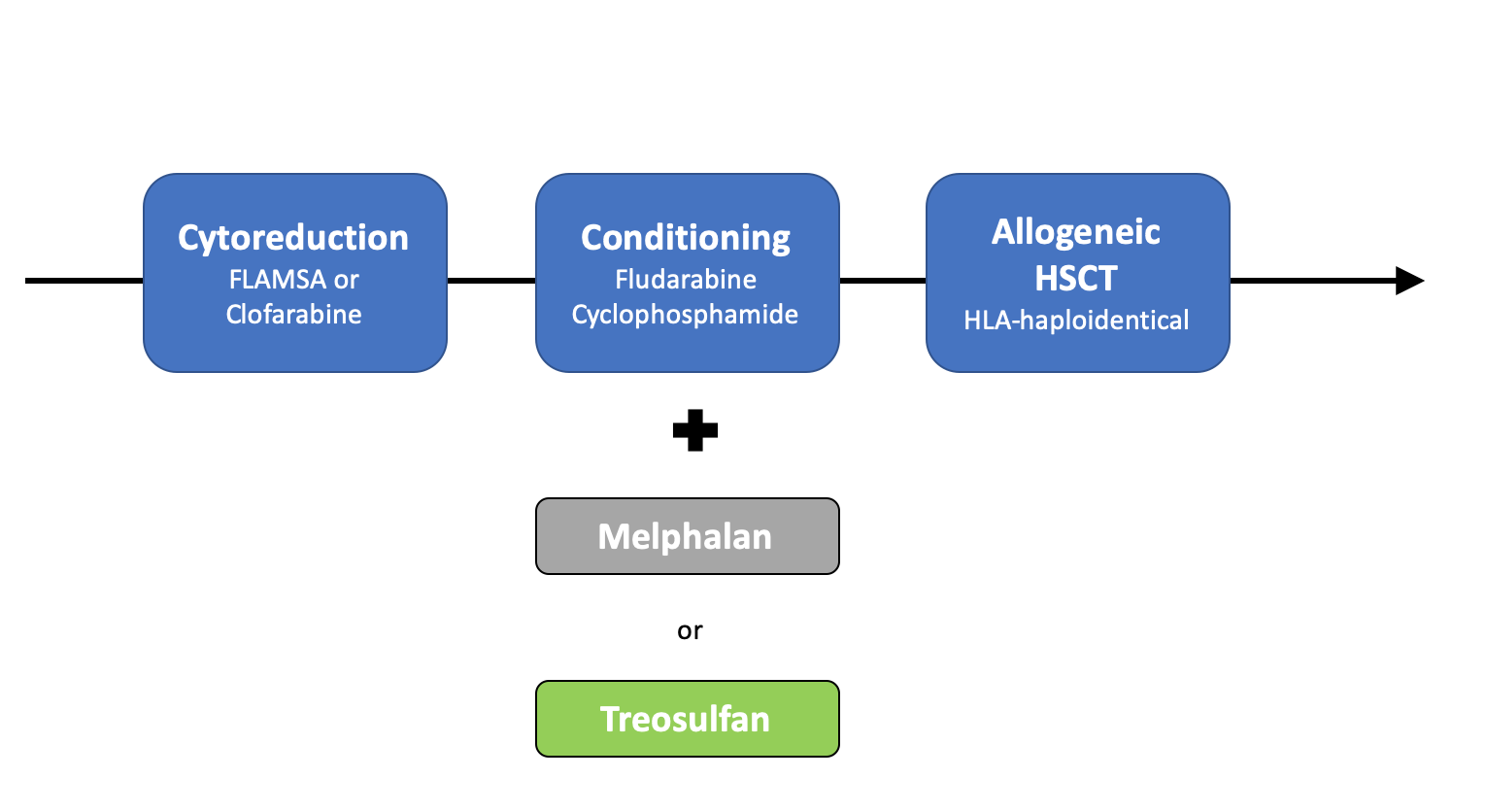

Supplement: Supplementary file 1 [file cancers-16-02859-s001.zip › Supplementary Figure S1.png]

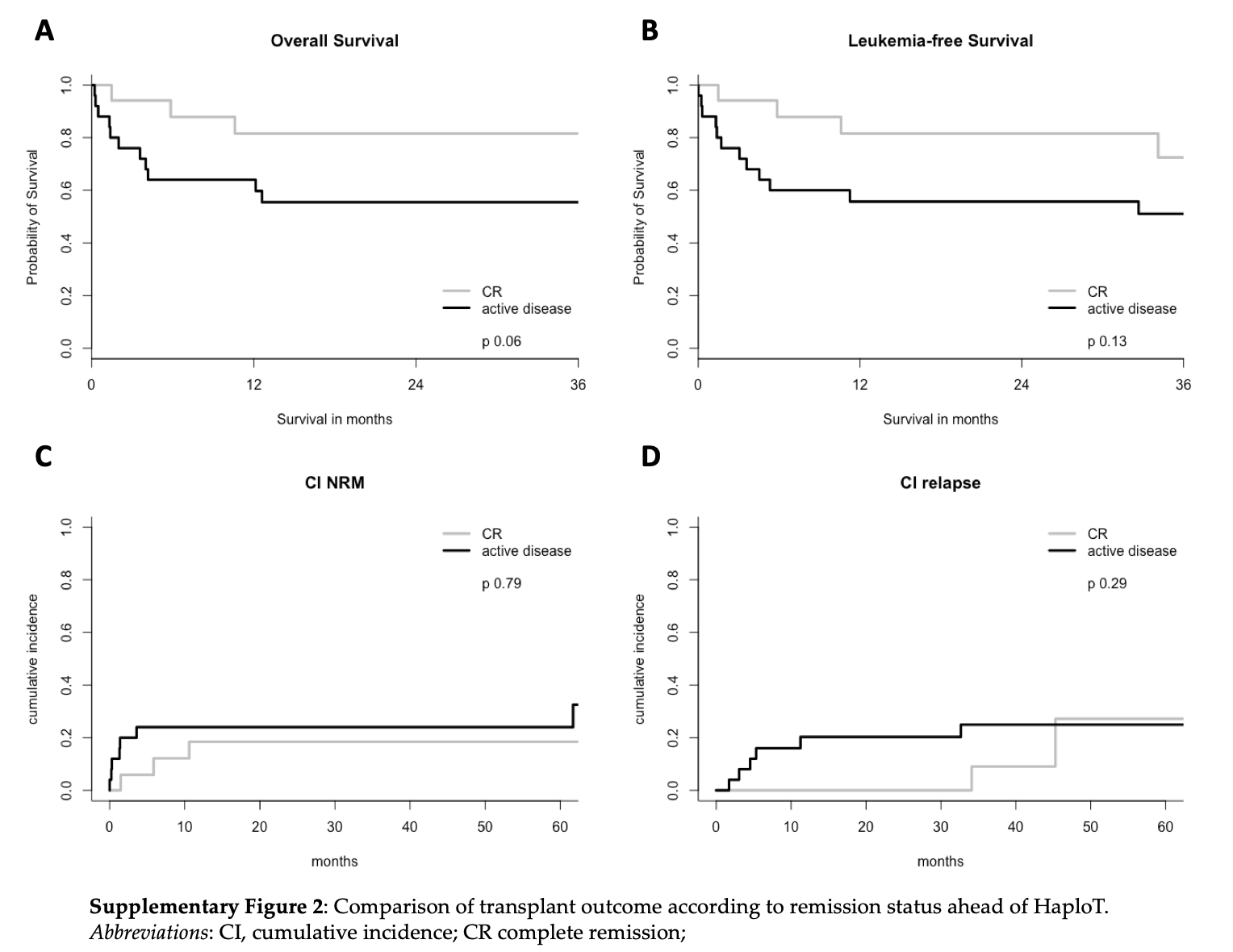

Supplement: Supplementary file 1 [file cancers-16-02859-s001.zip › Supplementary Figure S2.png]
